# Experimental Study of Aluminium-Timber Composite Bolted Connections Strengthened with Toothed Plates

**DOI:** 10.3390/ma15155271

**Published:** 2022-07-30

**Authors:** Marcin Chybiński, Łukasz Polus

**Affiliations:** Institute of Building Engineering, Faculty of Civil and Transport Engineering, Poznan University of Technology, Piotrowo 5 Street, 60-965 Poznan, Poland

**Keywords:** aluminium-timber composite structures, aluminium alloy, engineering wood products, laminated veneer lumber (LVL), toothed plate, bolted connection, shear connection, push-out test

## Abstract

This paper presents the first experimental study of the load-slip behaviour of aluminium-timber composite bolted connections reinforced with toothed plates. The effectiveness of the strengthening was evaluated in laboratory push-out tests. The push-out test samples consisted of laminated veneer lumber panels, aluminium alloy I-beams, and bolts (grade 8.8 10 mm × 125 mm and 12 mm × 135 mm bolts, grade 5.8 10 mm × 125 mm and 12 mm × 135 mm bolts). A group of 16 specimens had toothed plates as additional reinforcement, while 16 specimens had no reinforcement. The impact of the bolt diameter (10 and 12 mm) and bolt grade (5.8 and 8.8) on the behaviour of the connections was also analysed. The values of the ultimate load and the slip modulus for the bolted connections with grade 8.8 10 mm and 12 mm bolts and with grade 5.8 12 mm bolts reinforced by toothed-plate connectors were comparable to the values for the non-reinforced connections. This was because, in the case of grade 8.8 10 mm × 125 mm and 12 mm × 135 mm bolts and grade 5.8 12 mm × 135 mm bolts, the laminated veneer lumber (LVL) slabs split both in the reinforced and non-reinforced connections. The toothed-plate connectors reduced timber destruction in the bearing zones in the LVL slabs. However, they did not protect the LVL slabs against splitting. Therefore, the impact of the toothed plate connectors on the stiffness and strength of the bolted connections with grade 8.8 10 mm and 12 mm bolts and with grade 5.8 12 mm bolts analysed in this paper was found to be negligible. In the case of grade 5.8 10 mm bolts, the LVL slabs did not split. The mean slip modulus *k*_0.6_ of the connections with grade 5.8 10 mm bolts reinforced with toothed plate connectors was 2.9 times higher than that of the non-reinforced connections. However, the strength of the connections with grade 5.8 10 mm bolts was 1.2 times lower after reinforcing. This was because the shanks of the bolts were sheared faster in the reinforced connections than in the non-reinforced connections as a result of the bolt shanks being under the bearing pressure of the aluminium flange, the LVL slab, and the toothed-plate flange. This situation did not occur for the remaining connections because they had a higher strength (grade 8.8 bolts) or a larger diameter (12 mm), and their bolts were less prone to cutting off. The investigated load–slip curves of the reinforced bolted connections can be used for designing and numerical modelling of aluminium-timber composite beams with this type of connection.

## 1. Introduction

### 1.1. Literature Review

Modern designing requires the use of sustainable solutions and is open especially to those which can help reduce the carbon footprint, such as composite structural elements and composite materials. A composite structural element consists of at least two components made of different material and permanently joined with shear connectors (e.g., steel-concrete composite beams), whereas a composite material is a combination of materials with different properties (e.g., reinforced concrete or plywood). The use of composite structural elements is continuously on the rise due to the fact that it provides for increased load-bearing capacity and helps to overcome serviceability limitations [1]. For example, the load-bearing capacity of unrestrained aluminium beams increased 7.0 times after they were joined with timber slabs [2]. The stiffness of the aluminium beam analysed in [3] increased 4.3 times after it was used together with the timber slab in a composite beam. Greater stiffness leads to lower deflections. Due to this fact, the serviceability limit state (deflection ≤ limit deflection) for structural beams is easier to meet. The idea behind composite elements is to take the maximum advantage of the materials they are made of [4]. Similarly, composite materials are produced to form a material with properties different from the ones of the individual components. Increasingly more often, new composite materials, such as fibre-reinforced polymer composites or carbon-epoxy composites, have been used in modern design due to their high strength-to-weight ratio and durability [5,6,7]. Composite materials are often applied where traditional materials tend to fail [8]. Glass, aramid, or carbon fibre-reinforced polymer sheets are used to strengthen engineered wood products [9,10,11]. This is also an example of two different composite materials (laminated veneer lumber and fibre-reinforced polymer) used in one solution.

Recently, new composite structural elements with timber, such as timber-concrete composite elements [12,13,14], steel-timber composite beams [15,16,17] or aluminium-timber composite beams [18] have been investigated. Timber is one of the oldest known construction materials. Ancient Romans used it to build houses, temples, and bridges [19]. Timber has been used for many years to construct bridges and churches [20,21,22,23]. Structures made of timber can be easily repaired or reinforced [24,25,26]. Unfortunately, solid sawn timber has some limitations, e.g., in maximum lengths or cross-sectional dimensions. These limitations can be overcome by quality controlled engineered wood products, e.g., laminated veneer lumber (LVL) [27], cross banded laminated veneer lumber (LVL-C) [28], laminated strand lumber (LSL) [29], parallel strand lumber (PSL) [30], glued-laminated timber (GLT) [31] or cross-laminated timber (CLT) [32,33]. LVL or plywood were used for making slabs in aluminium-timber composite beams [34,35]. In addition to plywood and LVL, a slab may also be made of cross-laminated timber (CLT), which was demonstrated for steel-timber composite beams [36,37] (which are similar to aluminium-timber composite beams). An aluminium-timber composite beam is a sustainable example of a composite structure with a timber structural element. The use of aluminium alloy girders increases the durability of composite beams and reduces maintenance costs due to the light weight and high corrosion resistance of aluminium alloys [38]. The use of bolts as shear connectors makes it possible to demount composite beams at the end of their structural life in a way ensuring a sustainable use of natural sources. Aluminium-timber composite beams are lighter than steel-concrete composite beams and have replaceable parts. The lightness of aluminium-timber composite beams and the fact that there is no need to wait for the hardening of concrete may speed up the construction process. The aluminium alloy girders of aluminium-timber composite beams may have different cross-sections. For example, Saleh and Jasin [39] used rectangular hollow sections in their tests. Chybiński and Polus [2] investigated composite systems with extruded aluminium alloy I-girders. Girders may also be cold-formed, which was demonstrated for steel-timber composite beams [40], which are similar to aluminium-timber composite beams. For example, cold-rolled aluminium alloy members are fabricated in Australia [41]. The aluminium-timber composite beams presented in the literature have T-shaped cross-sections. Recently, Wang et al. [42,43] demonstrated that steel-timber composite beams may have I-shaped cross-sections. The steel-timber composite beams analysed by Wang et al. consisted of timber panels, U-shaped thin-walled steel beams, and bolts and screws as connectors. Aluminium-timber composite beams may have similar cross-sections. In each composite beam, connections play a crucial role and determine its behaviour. They should be ductile and have a characteristic slip capacity exceeding 6 mm [44]. They should also show high shear resistance and stiffness. It is advisable to reinforce connections to improve their mechanical parameters. When the shear resistance of a connection is higher, the lower number of connectors may be used to obtain a full composite action in a beam. When the stiffness of a connection is higher, the longitudinal slip between the slabs and the girders is lower.

### 1.2. Problem Statement and the Aim of the Current Research

In this paper, the authors analysed connections to be used in aluminium-timber composite beams. The load capacity of the connections with mechanical fasteners depends on many parameters, such as timber density, loading direction, fastener spacing, and end and edge distances [45]. The connections for aluminium-timber composite beams investigated in the previous studies were screwed or bolted [2,35,46,47,48,49]. Their stiffness and strength were relatively low. For this reason, Chybiński and Polus [49] proposed to use toothed plates in screwed connections as reinforcement. In case of screwed connections, the use of toothed plate connectors was found to be effective in increasing the strength of aluminium-timber composite connections. Enhancements of 35.0% (for Geka toothed-plate connectors and 12 mm screws), 28.7% (for Bulldog toothed-plate connectors and 10 mm screws), and 23.8% (for Bulldog toothed-plate connectors and 12 mm screws) were achieved. The use of toothed plate connectors did not have an impact on the stiffness of the screwed connections. Toothed-plate connectors were only used to reinforce screwed aluminium-timber connections in the aforementioned study [49]. In this paper, toothed plates were used to reinforce bolted aluminium-timber connections for the first time. The main aim of this paper is to determine the effectiveness of reinforcing bolted connection using toothed plates. The push-out laboratory tests on 16 reinforced and 16 non-reinforced samples were conducted to determine the shear resistance and stiffness of the bolted aluminium-timber connections. Moreover, the influence of the bolt diameter (10 and 12 mm) and bolt grade (5.8 and 8.8) on the mechanical parameters of the reinforced and non-reinforced connections was analysed. Finally, the behaviour of the bolted connections analysed in this paper was compared with the behaviour of the screwed connections analysed in the literature.

## 2. Materials and Methods

### 2.1. Aluminium Alloy

The extruded aluminium alloy I-beams were made of the AW-6060 T6 aluminium alloy. The yield strength, tensile strength, and Young’s modulus of this alloy were 181.5 MPa, 209.8 MPa, and 66,400 MPa, respectively [50].

### 2.2. LVL

Laminated veneer lumber (LVL) is made by laminating thin (3–4 mm) wood veneers using adhesives [51]. Veneers are peeled off of high-quality logs. They are oriented in a grain direction. LVL is now fabricated in the United States, Australia, Japan, New Zealand, Finland, and Poland. For the purpose of this study, STEICO LVL manufactured in Poland from Scots pine (*Pinus sylvestris* L.) and Norway spruce (*Picea abies* L. H. Karst) was used [52]. The compression strength (parallel to grain), tension strength (parallel to grain), bending strength (flatwise, parallel to grain), and Young’s modulus of this engineering wood product declared by the manufacturer were 40.0 MPa, 36.0 MPa, 50.0 MPa, and 14,000 MPa, respectively [53].

### 2.3. Bolts

Grade 8.8 10 mm × 125 mm and 12 mm × 135 mm bolts as well as grade 5.8 10 mm × 125 mm and 12 mm × 135 mm bolts were used as shear connectors. The length of the unthreaded shanks was the same in the 10 mm bolts and in the 12 mm bolts (90 mm). The characteristic yield strength *f_yb_* and the ultimate strength *f_ub_* of the bolts can be determined based on the bolt grade, e.g., in case of grade 8.8 bolt the ultimate strength is 800 MPa and the yield strength is 640 MPa. To confirm the bolt grade, the yield strength and the ultimate strength of the bolts were also evaluated experimentally in accordance with [54] in tensile tests. The tests were carried out using an Instron 4483 testing machine (Instron, Grove City, PA, USA). The mechanical parameters of two bolts per each bolt type were investigated.

### 2.4. Toothed Plates

**Figure 1:**
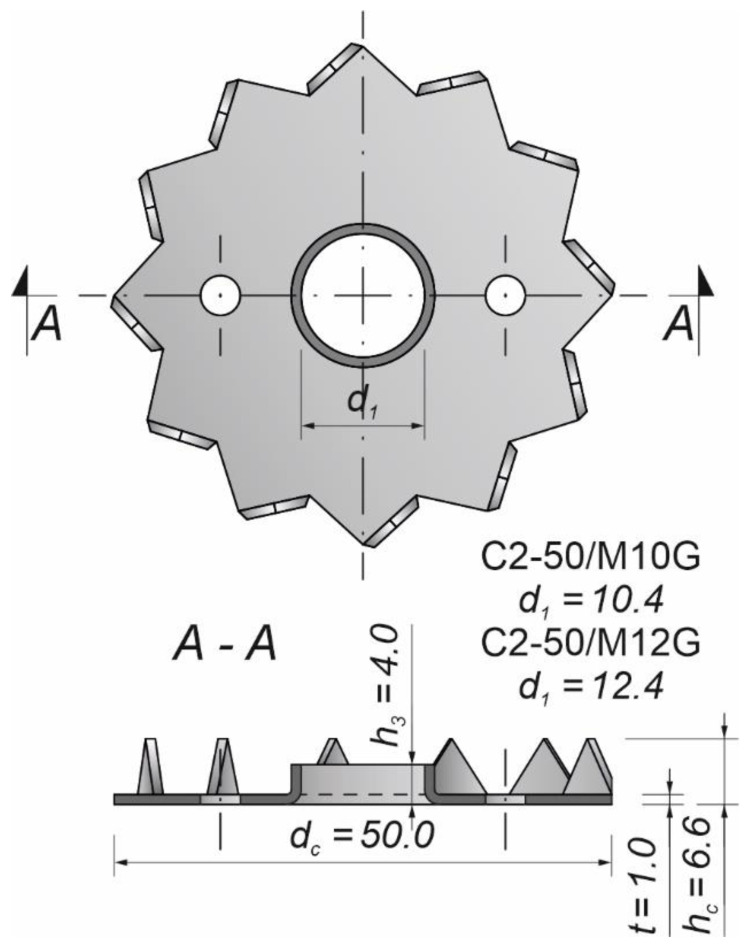
C2 (Bulldog) toothed-plate connector.

Bulldog toothed-plate connectors (C2-50/M10G and C2-50/M12G) were used for reinforcing aluminium-timber bolted connections (see Figure 1). The names of the toothed plates contained information on 4 parameters: C2 represented the toothed plate type, 50 was the plate diameter in mm, M10 or M12 were the types of bolts suggested for use with the plate, and G meant that the plates were galvanised [55].

### 2.5. Push-Out Tests

**Figure 2:**
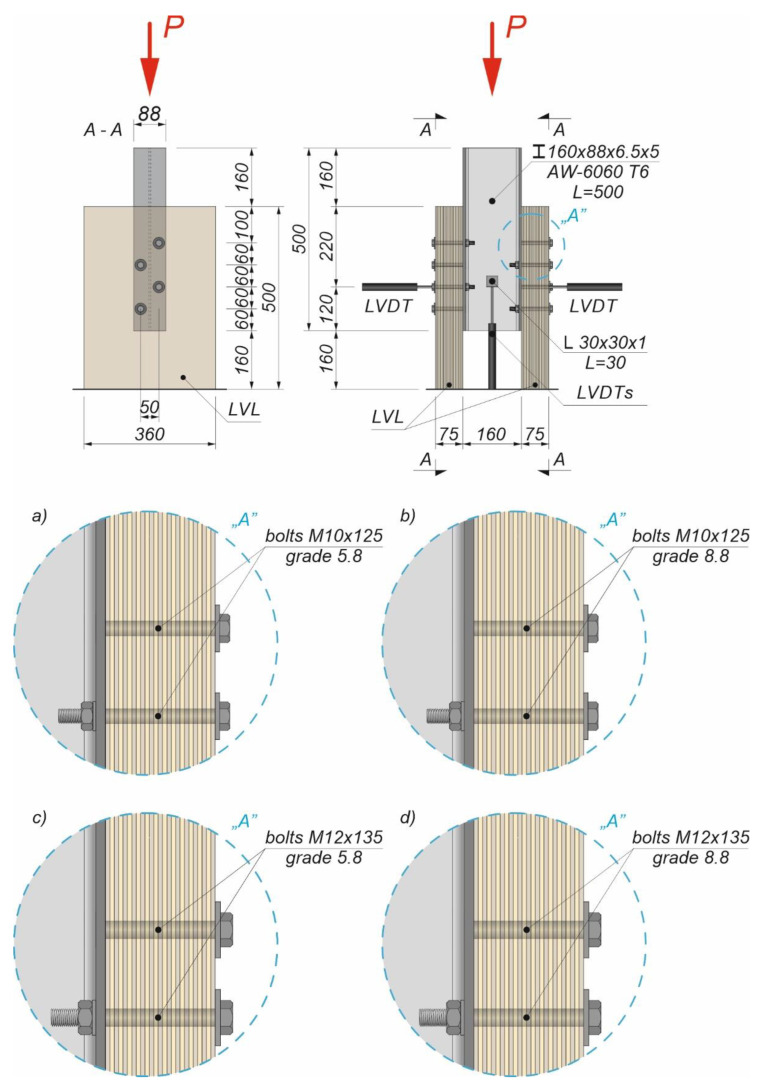
The tested specimens: (**a**) with 10 mm 5.8 grade bolts and without reinforcing toothed plates; (**b**) with 10 mm 8.8 grade bolts and without reinforcing toothed plates; (**c**) with 12 mm 5.8 grade bolts and without reinforcing toothed plates; and (**d**) with 12 mm 8.8 grade bolts and without reinforcing toothed plates.

**Figure 3:**
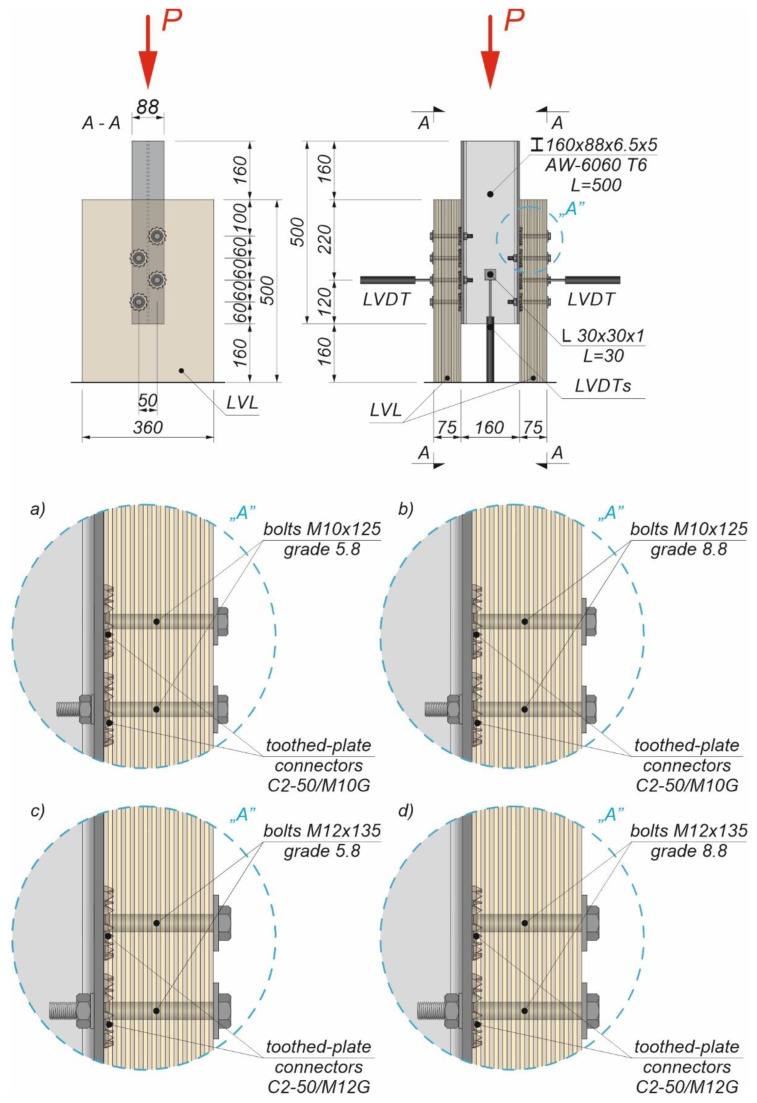
The tested specimens: (**a**) with 10 mm 5.8 grade bolts and reinforcing C2-50/M10G toothed plates; (**b**) with 10 mm 8.8 grade bolts and reinforcing C2-50/M10G toothed plates; (**c**) with 12 mm 5.8 grade bolts and reinforcing C2-50/M12G toothed plates; and (**d**) with 12 mm 8.8 grade bolts and reinforcing C2-50/M12G toothed plates.

An Instron 8505 Plus machine (Instron, HighWycombe, Buckinghamshire, UK) was used to investigate the load–slip behaviour of 32 specimens. Each specimen consisted of two LVL panels and an extruded aluminium alloy I-beam (see Figure 2 and Figure 3).

The LVL panels were connected with the extruded aluminium alloy I-beams using eight variants of connections presented in Table 1.

The holes in the extruded aluminium alloy I-beams and LVL panels had the same diameters as the bolts to reduce the slip between the upper aluminium girder flange and the LVL panel. In each specimen, the bolts were installed using a torque wrench (Sandvik Belzer, IZO-I-100, 10–100 Nm) (Sandvik, Portlaoise, Ireland). The loading direction was parallel to the LVL grain, and the tread–grain angle was 90°. The torque level was measured during the installation of the bolts using a torque wrench and recorded at the end of the installation process (35 Nm for the 10 mm bolt, 60 Nm for the 12 mm bolt). Before the tests, the toothed plates were pressed into the LVL panels using a compressive force of 35 kN generated by a hydraulic press. The bolts were evenly spaced out (the space between the bolts was 50 mm in the transverse direction and 60 mm in the longitudinal direction). Staggered spacing was applied to avoid the overlapping of toothed plates. The slip between the LVL panels and the extruded aluminium alloy I-beam and the horizontal move of the sample were measured using linear variable differential transformers (LVDTs) (Figure 2 and Figure 3).

The push-out tests were conducted in accordance with [56]. A load control regime was used during the first stage of the tests to obtain a regular shape of the shear force–slip curve and to read the slip modulus of the connection for a load equal to 40% and 60% of the maximum value of the force. The constant rate of displacement was applied during the second stage of the tests. Due to this fact, the behaviour of the connections after the maximum load had been achieved could be observed. First, the load was increased from 0 to 40% of the estimated force over 2 min. Next, it remained at this level for 30 s. Subsequently, the load was decreased from 40% to 10% of the estimated force and maintained at this level for 30 s. Subsequently, the value of the load was increased from 10% to 70% of the estimated force. Up to that point, the test was conducted using a load control regime. From then on, it was conducted using a displacement control regime (5.0 mm/min). The estimated force of 195.2 kN was calculated taking into account eight bolts and the ultimate load per one M10 bolt (24.4 kN) obtained in the previous test presented in [2]. The value of the estimated force as well as the loading procedure were modified during the tests based on the previous results.

## 3. Results

### 3.1. Tensile Tests Results

The yield strength and the tensile strength of the bolts used in this study are presented in Table 2.

### 3.2. Shear Connection Tests Results

**Figure 4:**
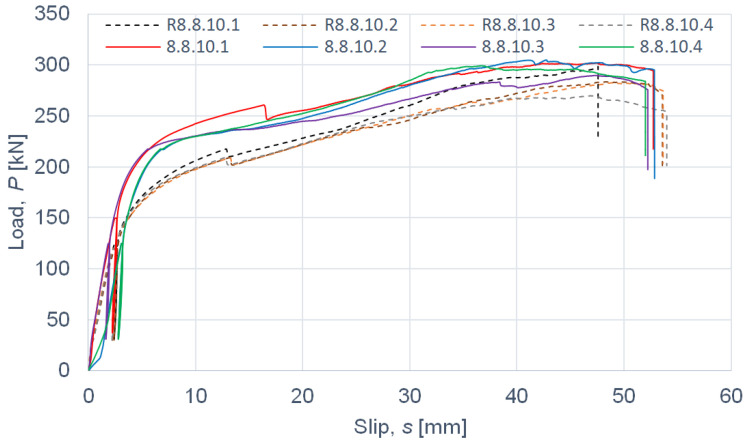
The load–slip curves from the push-out tests of the shear connections with 10-mm grade 8.8 bolts and with toothed-plate connectors (type C2-50/M10G, Bulldog) in specimens 8.8.10.1–8.8.10.4 or without toothed-plate connectors in specimens R8.8.10.1–R8.8.10.4.

**Figure 5:**
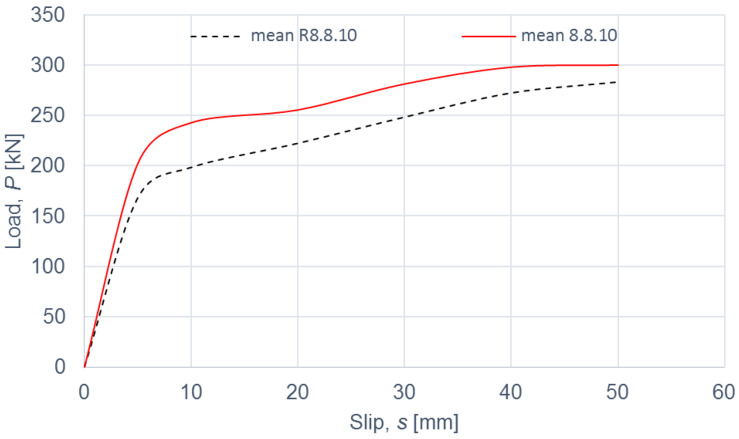
The mean load–slip curves for specimens 8.8.10.1–8.8.10.4 and R8.8.10.1–R8.8.10.4.

**Figure 6:**
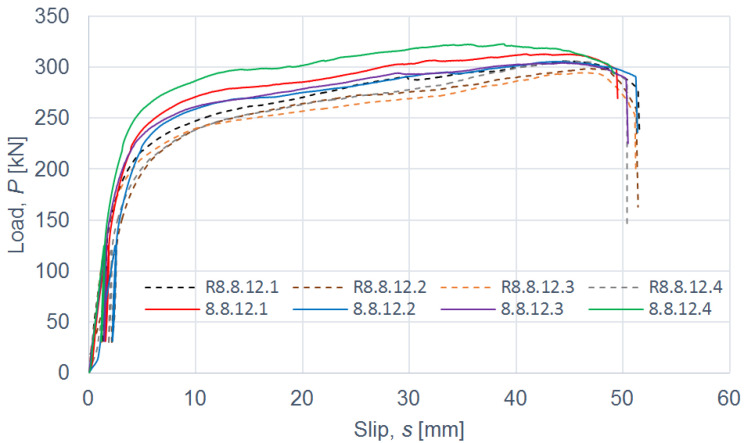
The load–slip curves from the push-out tests of the shear connections with 12-mm grade 8.8 bolts and with toothed-plate connectors (type C2-50/M12G, Bulldog) in specimens 8.8.12.1–8.8.12.4 or without toothed-plate connectors in specimens R8.8.12.1–R8.8.12.4.

**Figure 7:**
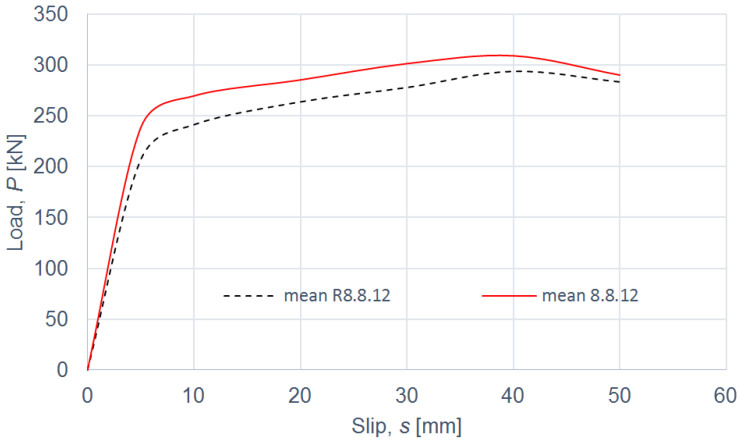
The mean load–slip curves for specimens 8.8.12.1–8.8.12.4 and R8.8.12.1–R8.8.12.4.

**Figure 8:**
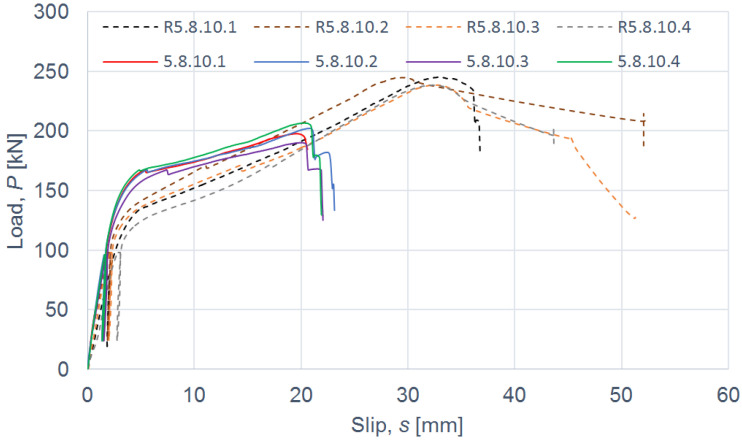
The load–slip curves from the push-out tests of the shear connections with 10-mm grade 5.8 bolts and with toothed-plate connectors (type C2-50/M10G, Bulldog) in specimens 5.8.10.1–5.8.10.4 or without toothed-plate connectors in specimens R5.8.10.1–R5.8.10.4.

**Figure 9:**
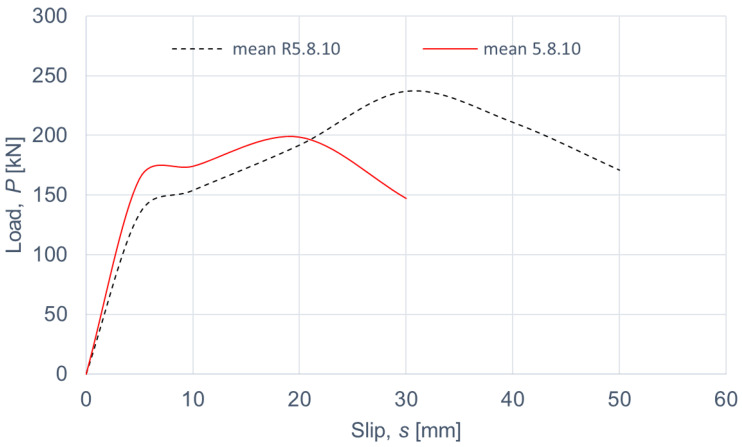
The mean load–slip curves for specimens 5.8.10.1–5.8.10.4 and R5.8.10.1–R5.8.10.4.

**Figure 10:**
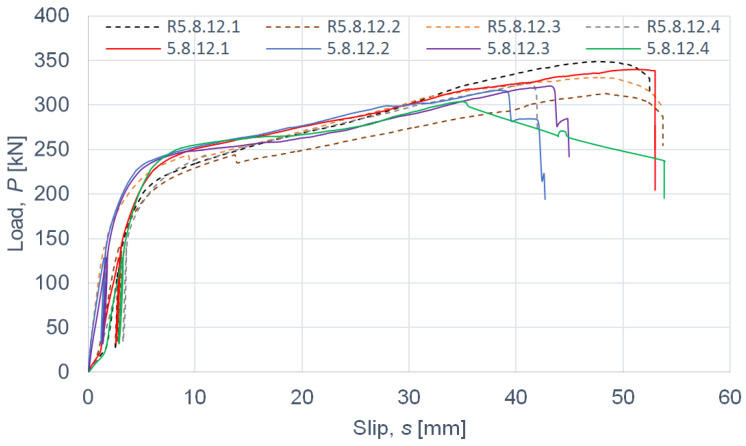
The load–slip curves from the push-out tests of the shear connections with 12-mm grade 5.8 bolts and with toothed-plate connectors (type C2-50/M12G, Bulldog) in specimens 5.8.12.1–5.8.12.4 or without toothed-plate connectors in specimens R5.8.12.1–R5.8.12.4.

**Figure 11:**
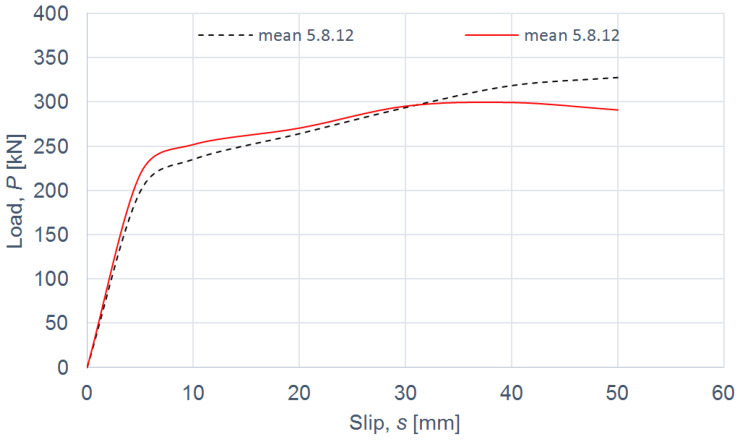
The mean load–slip curves for specimens 5.8.12.1–5.8.12.4 and R5.8.12.1–R5.8.12.4.

The load–slip curves from the push-out tests are presented in Figures 4, 6, 8 and 10. The mean load–slip curves for each connection variant are shown in Figures 5, 7, 9 and 11 to describe the behavior of each variant in a simplified manner. The ultimate load per one connector (*P_ult_*), the value of the slip corresponding to the ultimate load (*s_ult_*), and the slip moduli per one connector (*k*_0.4_ and *k*_0.6_) are shown in Table 3, Table 4, Table 5, Table 6, Table 7, Table 8, Table 9 and Table 10. The measurement errors for values presented in Table 3, Table 4, Table 5, Table 6, Table 7, Table 8, Table 9 and Table 10 were calculated using a Student’s t-distribution with three degrees of freedom and a confidence level of 0.95. The slip modulus *k*_0.4_ per one connector was calculated as the ratio of 40% of the ultimate load per one connector to the slip corresponding to this value of the load. The slip modulus *k*_0.4_ may be used for serviceability limit state calculations [58]. The slip modulus *k*_0.6_ per one connector was calculated as the ratio of 60% of the ultimate load per one connector to the slip corresponding to this value of the load. The slip modulus *k*_0.6_ may be used for the ultimate limit state calculations [59].

In the case of grade 8.8 10 mm bolts, the values of the ultimate load per one connector and of the slip modulus per one connector *k*_0.4_ for the specimens with toothed-plate connectors were comparable to the values for the specimens without toothed-plate connectors (compare Table 3 and Table 4).

The mean value of the slip modulus per one connector *k*_0.6_ for the specimens with toothed-plate connectors was insignificantly higher than for the specimens without toothed-plate connectors (compare Table 3 and Table 4). The energy accumulated in specimens 8.8.10.1–8.8.10.4 with toothed-plate connectors was higher than in specimens R8.8.10.1–R8.8.10.4 without toothed-plate connectors, due to the fact that all the load–slip curves of the reinforced connections had a higher load than the non-reinforced connections for the same displacement (see Figure 4 and Figure 5).

In the case of grade 8.8 12-mm bolts, the same conclusions can be drawn as for grade 8.8 10-mm bolts (see Table 5 and Table 6 and Figure 6 and Figure 7). Upon comparing the load-carrying capacities and the slip moduli of the tested connections with grade 8.8 bolts, it was observed that the use of toothed plate connectors was ineffective in improving both the load-carrying capacity and the stiffness of aluminium-timber composite connections.

The connections with grade 5.8 10-mm bolts reinforced with toothed plate connectors showed higher stiffness (*k*_0.6_ = 6.9 kN/mm per one connector) than the non-reinforced connections (*k*_0.6_ = 2.4 kN/mm per one connector) (compare Table 7 and Table 8). However, their strength was 1.2 times lower after reinforcing (see Figure 8 and Figure 9). After comparing the behaviour of the non-reinforced connections and the reinforced connections with grade 5.8 10-mm bolts, the following conclusions may be drawn. The toothed-plate connectors reduced timber destruction in the bearing zones in the LVL slabs, because some part of the load was transferred by the teeth. However, the shanks of the bolts were sheared faster in the reinforced connections due to the fact that the bolt shanks were under the bearing pressure from the aluminum flange and the LVL slab as well as the toothed-plate flange. In the case of non-reinforced connections, the bolts were more tensioned than sheared, whereas in the case of reinforced connections, it was the opposite. For these reasons, the reinforced connections were both stiffer and weaker than the non-reinforced connections with grade 5.8 10-mm bolts. The above did not occur for the remaining connections because they had a higher strength (grade 8.8 bolts) or a larger diameter (12 mm). Additionally, the LVL slabs were split in the reinforced and non-reinforced connections with grade 8.8 10 mm × 125 mm and 12 mm × 135 mm bolts, and grade 5.8 12 mm × 135 mm bolts.

In the case of grade 5.8 12-mm bolts, it was observed that the use of toothed plate connectors was ineffective in improving both the load-carrying capacity and the stiffness of aluminium-timber composite connections (see Table 9 and Table 10 and Figure 10 and Figure 11).

Connections are ductile if their characteristic slip capacity is at least 6 mm [60]. The value of the characteristic slip capacity exceeded 6 mm in all tested connections, and therefore they were all ductile.

**Figure 12:**
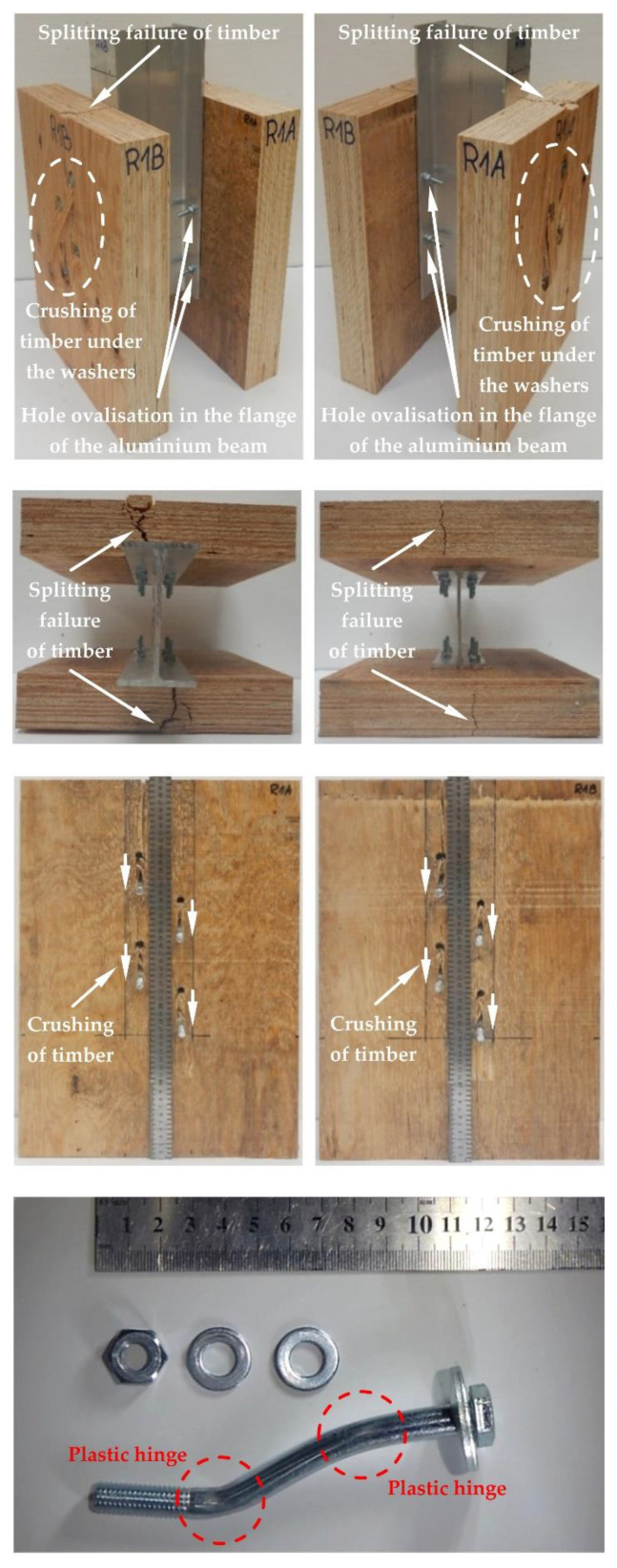
The failure mode of the aluminium-timber connections with 10-mm 8.8 grade bolts and without reinforcing toothed plates.

**Figure 13:**
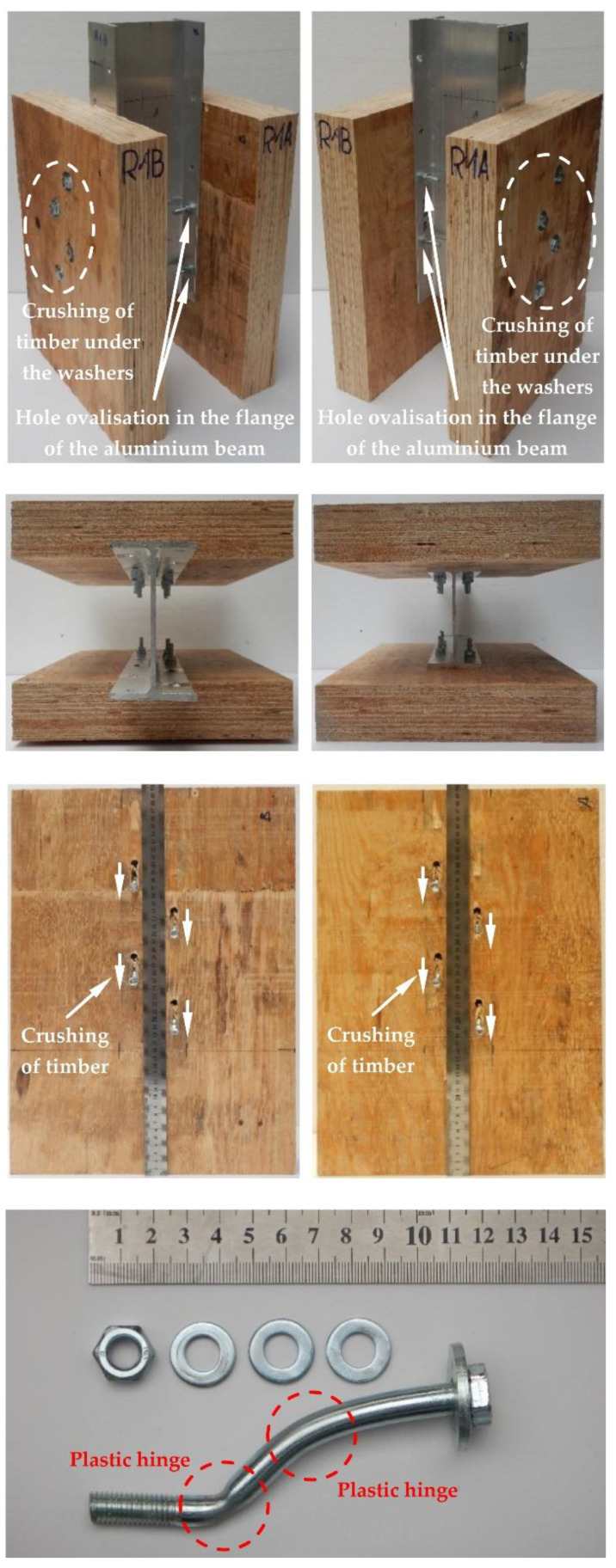
The failure mode of the aluminium-timber connections with 10-mm 5.8 grade bolts and without reinforcing toothed plates.

**Figure 14:**
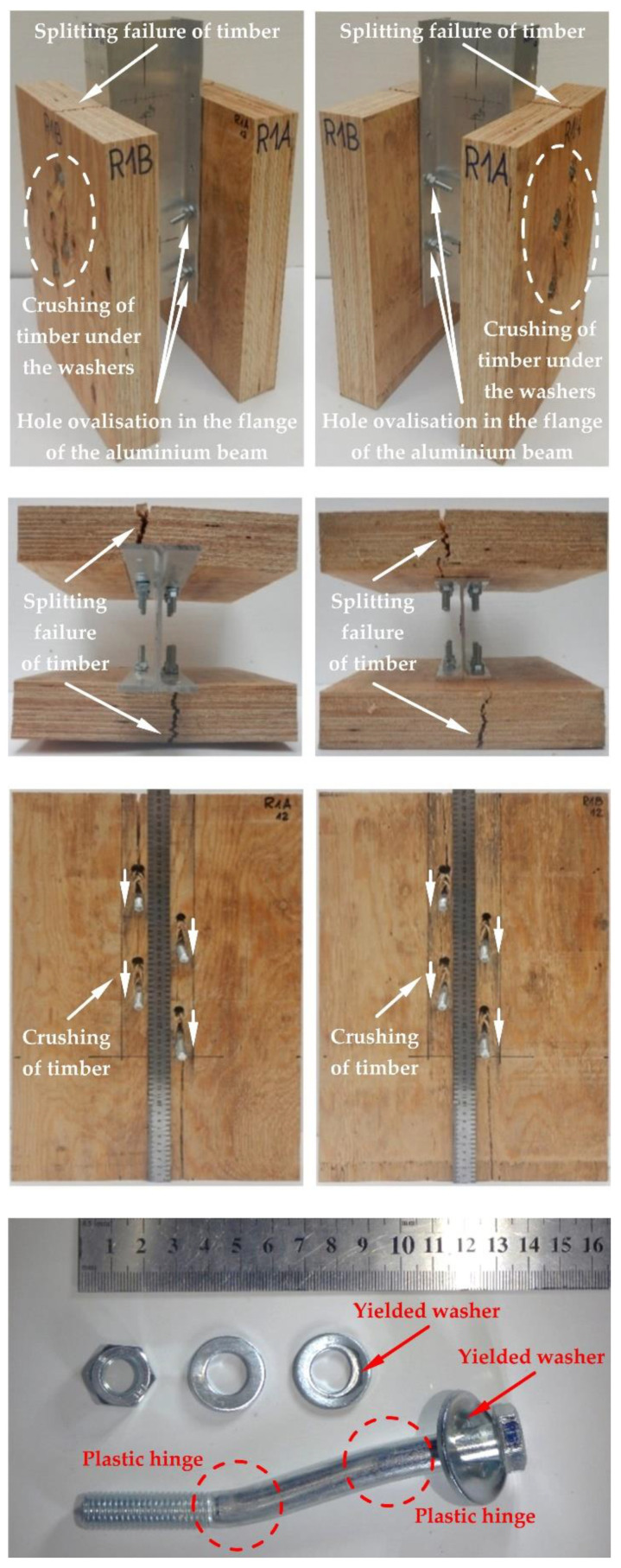
The failure mode of the aluminium-timber connections with 12-mm 8.8 grade bolts and without reinforcing toothed plates.

**Figure 15:**
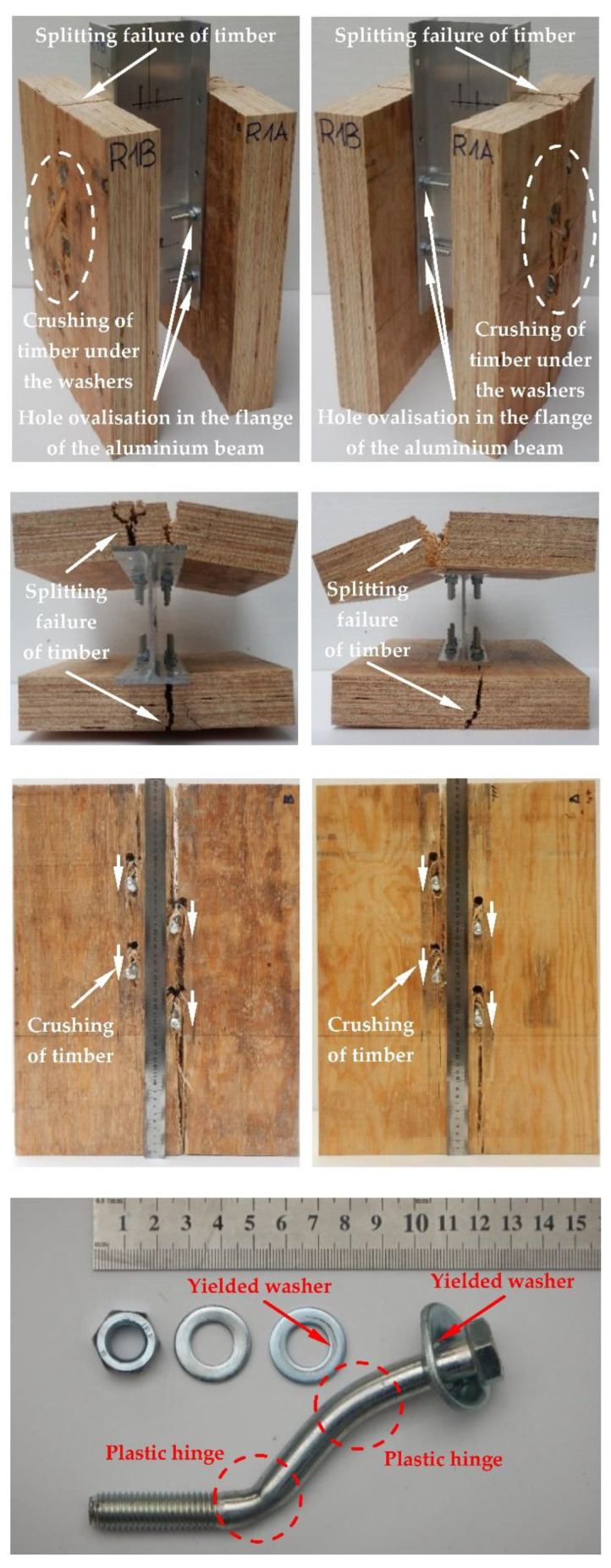
The failure mode of the aluminium-timber connections with 12-mm 5.8 grade bolts and without reinforcing toothed plates.

**Figure 16:**
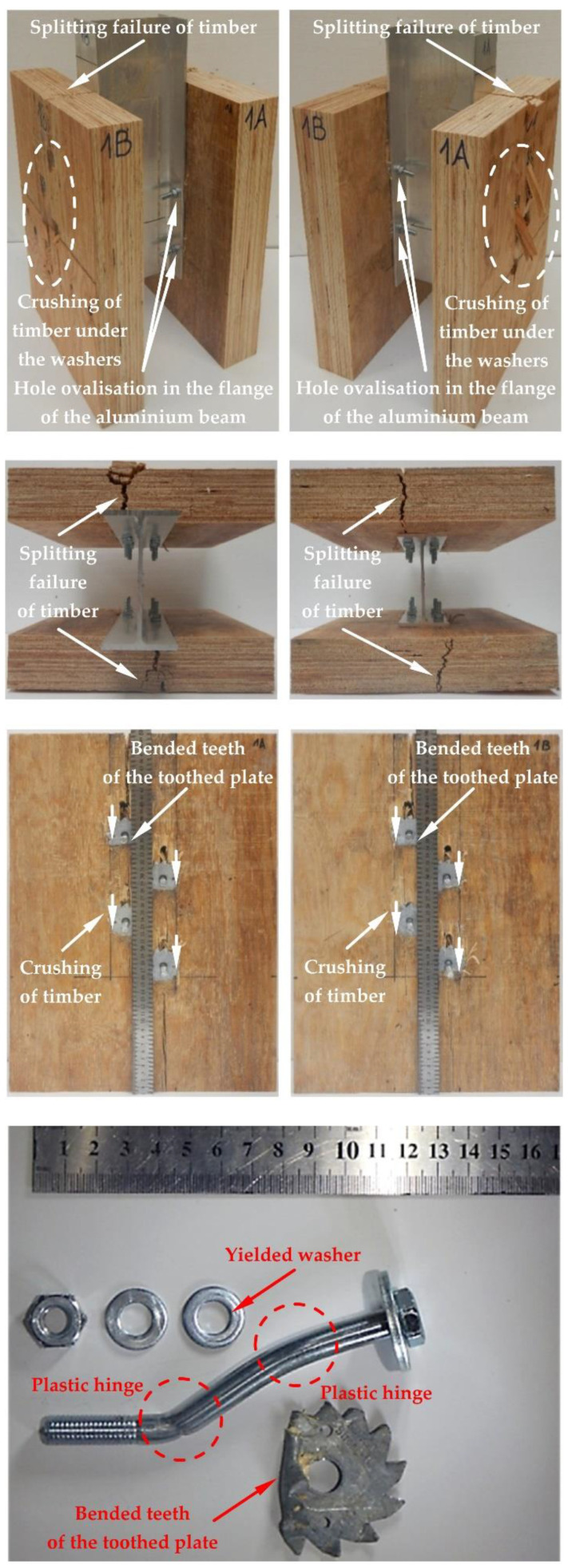
The failure mode of the aluminium-timber connections with 10-mm 8.8 grade bolts and with reinforcing toothed plates (type C2-50/M10G, Bulldog).

**Figure 17:**
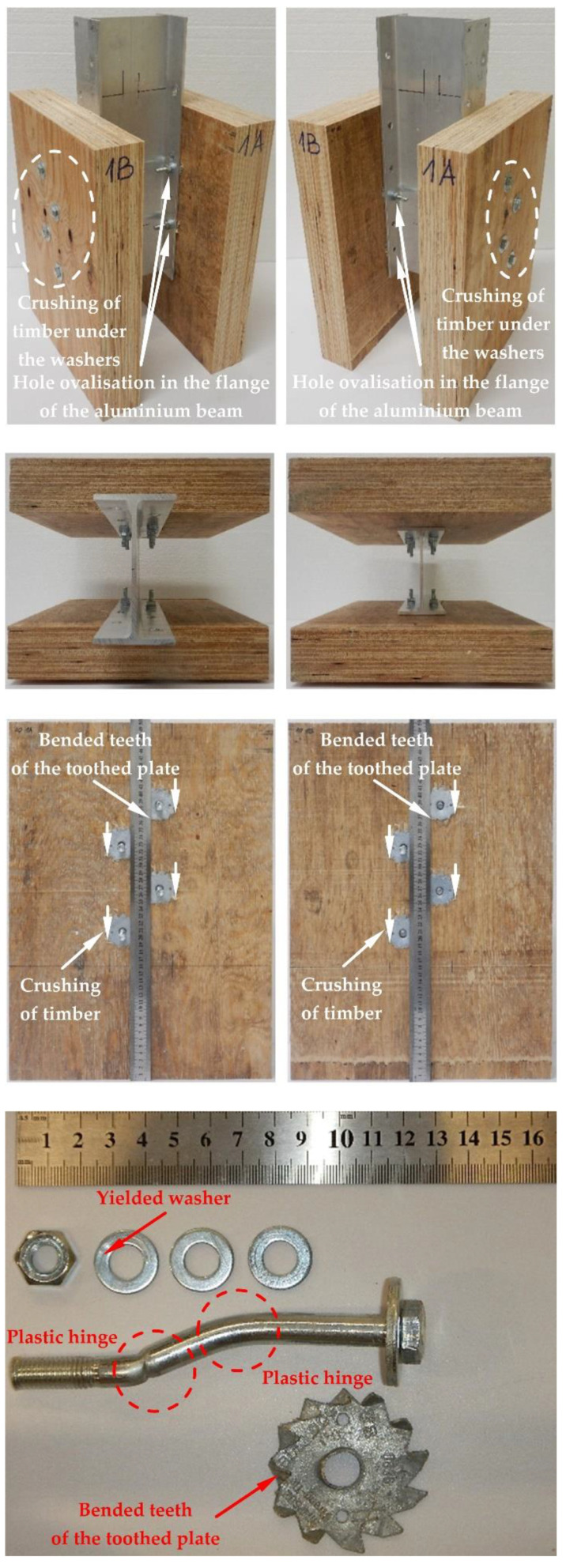
The failure mode of the aluminium-timber connections with 10-mm 5.8 grade bolts and with reinforcing toothed plates (type C2-50/M10G, Bulldog).

**Figure 18:**
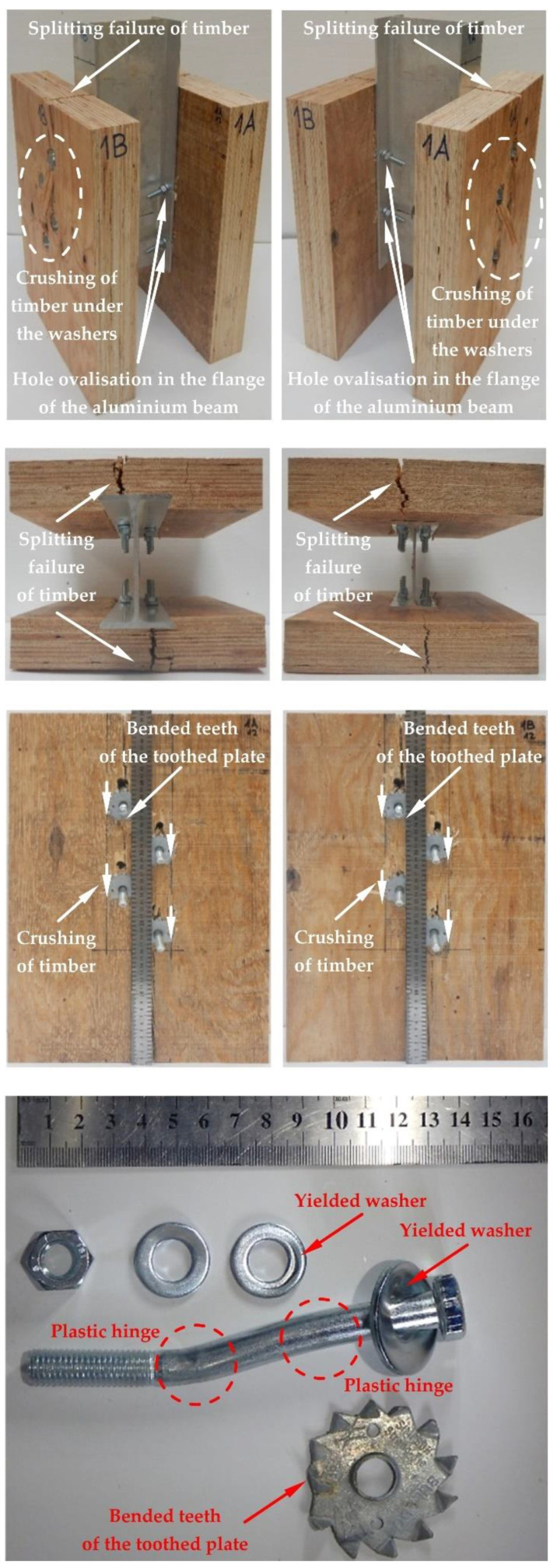
The failure mode of the aluminium-timber connections with 12-mm 8.8 grade bolts and with reinforcing toothed plates (type C2-50/M12G, Bulldog).

**Figure 19:**
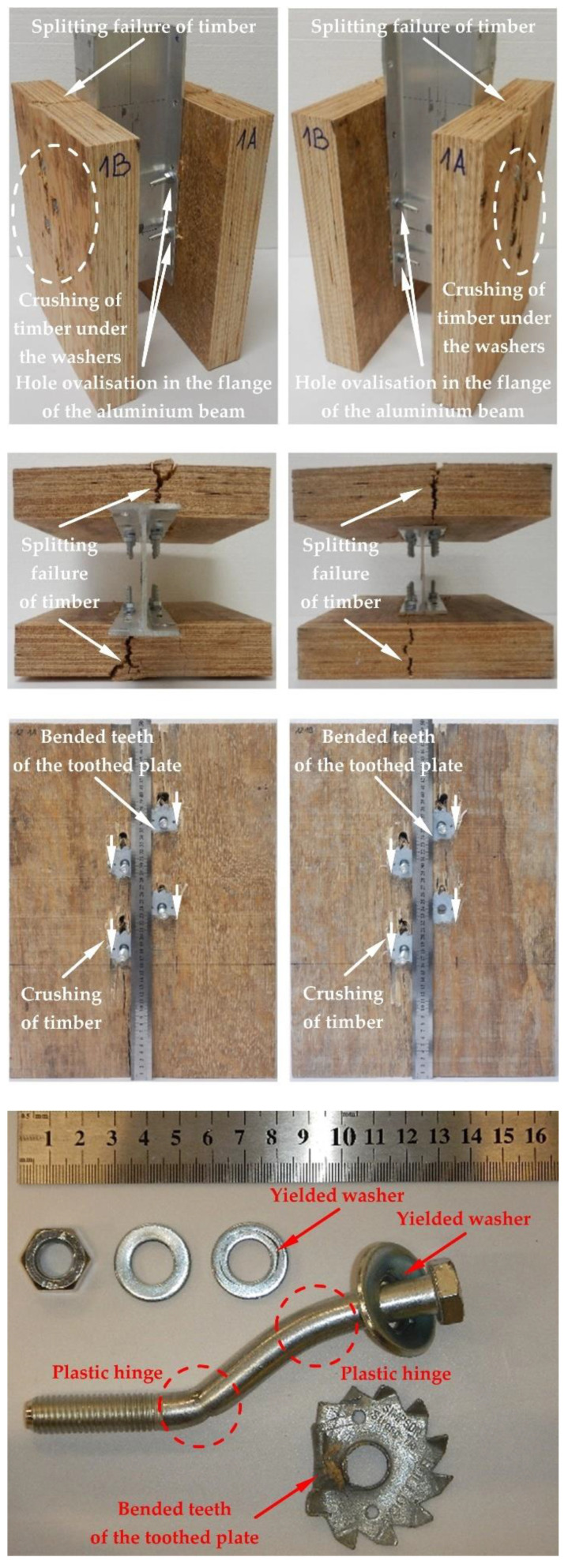
The failure mode of the aluminium-timber connections with 12-mm 5.8 grade bolts and with reinforcing toothed plates (type C2-50/M12G, Bulldog).

The modes of failure of the tested bolted connections with or without Bulldog toothed-plate connectors (C2-50/M10G, C2-50/M12G) are presented in Figure 12, Figure 13, Figure 14, Figure 15, Figure 16, Figure 17, Figure 18 and Figure 19. The authors observed the formation of two plastic hinges within the bolt, the crushing of LVL near the bolts, hole ovalisation in the flange of the aluminium alloy beam, bent teeth of toothed plates, and yielded washers due to washer pressure. Some of the bolts were additionally sheared. Furthermore, the LVL slabs were split both in the reinforced and non-reinforced connections with grade 8.8 10 mm × 125 mm and 12 mm × 135 mm bolts, and grade 5.8 12 mm × 135 mm bolts. This also explains why reinforcing was ineffective for the connections with these bolts. The LVL slabs in the connections with 5.8 10 mm × 125 mm bolts were not split.

## 4. Discussion

### 4.1. A Comparison of the Obtained Results with the Literature

In this paper, bolts were used to connect LVL slabs with aluminium beams. Another option is to use screws as shear connectors [49]. The push-out samples with bolts analysed in this article had the same geometry as the push-out samples with screws investigated in [49]. The screws had the same diameter and grade as the bolts. Moreover, the screwed connections were reinforced using the same toothed plates as the bolted connections. This made it possible to compare the structural behaviour of such connections (Table 11).

In the case of the screwed connections, the strength increased 1.3 times (10 mm) or 1.2 times (12 mm) after reinforcing. The splitting of timber was not observed. The use of toothed-plate connectors reduced timber destruction in the bearing zones and provided for a strength increase. In the case of the bolted connections with grade 5.8 12-mm bolts, the strength hardly changed after reinforcing. The connection strength was limited by the splitting strength of timber, and the toothed-plate connectors did not protect the timber slabs against splitting. In the bolted connections, the washers located on the LVL panels prevented the withdrawal of the bolts, and the LVL panels were split by the bolt shanks (“knife effect”). For this reason, the LVL panels in the bolted connections were more prone to splitting than in the screwed connections in which the withdrawal was prevented by the screw threads. In the case of the bolted connections with grade 5.8 10-mm bolts, the strength was 1.2 times lower after reinforcing. In these connections, the bolt shanks were under the bearing pressure of the aluminium flange and the LVL slab as well as the toothed-plate flange, and the bolts were sheared faster in the reinforced connections. This situation did not occur for the bolted connections with grade 8.8 bolts and with bolts with a larger diameter (12 mm), as they had higher shear resistance. The strength of the connections with grade 5.8 10-mm bolts was limited by the shear resistance of the bolts. Moreover, the grade 5.8 10-mm bolts also demonstrated a lower ultimate strength (483 MPa) than the 5.8 10-mm screws (553.9 MPa) [49], and they were more prone to cutting off during the push-out tests. For these reasons, in the connections with grade 5.8 10-mm bolts, the LVL slabs did not split. Both for the screwed and the bolted connections, the increase of the connector diameter provided for the increase of the connection shear strength. The non-reinforced bolted connections had a 1.8 times higher shear strength than the non-reinforced screwed connections.

### 4.2. Future Research and Possible Applications of the Results of This Study

The behaviour of composite beams depends on the mechanical parameters of their connections. The results of the double-shear tests presented in this study, such as shear resistance, can be used to evaluate the number of connectors necessary to achieve the full composite action in beams. Moreover, the obtained load–slip curves from the laboratory push-out tests can be used in finite element models of aluminium-timber composite beams to model the behaviour of connections which are discrete and represented by spring elements. Finite element analyses may be used to complement laboratory tests. Models with spring elements can reflect the behaviour of a real structure [61]. Moreover, spring elements provide for high computational speed. Discrete modelling of connections was used for example in the numerical models presented in [62,63,64].

Further experimental shear connection tests should be performed. The impact of the shear connector length on its strength may be examined. The influence of the toothed-plate connectors on the behaviour of the connections, the strength of which does not depend on the splitting strength of LVL (e.g., connections with bolts of a smaller diameter or of wider spacing than those used in this study), is still worth analysing.

## 5. Conclusions

In this paper, eight groups of push-out tests were conducted to investigate the load-carrying capacity, stiffness, load–slip response, failure modes, and ductility of aluminium-timber bolted connections strengthened or unstrengthened with toothed plates.

The results of the double-shear tests conducted in the study lead to some important conclusions. The toothed plate connectors were found ineffective in improving the stiffness and the strength of the bolted connections with grade 8.8 10 mm × 125 mm and 12 mm × 135 mm bolts, and grade 5.8 10 mm × 125 mm and 12 mm × 135 mm bolts. In the case of grade 8.8 10 mm × 125 mm and 12 mm × 135 mm bolts and grade 5.8 12 mm × 135 mm bolts, the LVL slabs split both in the reinforced and non-reinforced connections. The toothed-plate connectors reduced timber destruction in the bearing zones in the LVL slabs, but they did not protect the LVL slabs against splitting. The ultimate load of the connections with grade 8.8 10 mm × 125 mm and 12 mm × 135 mm bolts and grade 5.8 12 mm × 135 mm bolts mainly depended on the splitting strength of timber. In the case of grade 5.8 10-mm bolts, the LVL slabs did not split. The reinforced connections were both stiffer and weaker than the non-reinforced connections with grade 5.8 10-mm bolts. This was because the shanks of the bolts were sheared faster in the reinforced connections than in the non-reinforced connections. The reason for this was that the bolt shanks were under the bearing pressure of the aluminium flange, the LVL slab, and the toothed-plate flange. This situation did not occur for the remaining connections because they had a higher strength (grade 8.8 bolts) or a larger diameter (12 mm).

The results of the push-out tests of the bolted connections were compared with the results of the shear connection tests of the screwed connections presented in [49]. The non-reinforced bolted connections had 1.8 times higher shear strength than the non-reinforced screwed connections. In the case of the screwed connections, the reinforcing with toothed-plate connectors provided for a shear strength increase, whereas in the case of the bolted connections, it did not, as their strength was limited by the splitting strength of timber.

## Figures and Tables

**Table 1:** The variants of connections analysed in this study.

Variant	Designation of Specimens [mm]	Bolt Diameter and Length [mm]	Bolt Grade	Designation of a Reinforcing Toothed Plate
1	R8.8.10.1–R8.8.10.4	10 × 125	8.8	–
2	8.8.10.1–8.8.10.4	10 × 125	8.8	C2-50/M10G
3	R8.8.12.1–R8.8.12.4	12 × 135	8.8	–
4	8.8.12.1–8.8.12.4	12 × 135	8.8	C2-50/M12G
5	R5.8.10.1–R5.8.10.4	10 × 125	5.8	–
6	5.8.10.1–5.8.10.4	10 × 125	5.8	C2-50/M10G
7	R5.8.12.1–R5.8.12.4	12 × 135	5.8	–
8	5.8.12.1–5.8.12.4	12 × 135	5.8	C2-50/M12G

**Table 2:** The results of the tensile tests of the bolts (*f_yb_*—the mean value of the yield strength of the bolts from two tests, *f_ub_*—the mean value of the tensile strength of the bolts from two tests) [57].

Parameter	Bolt			
	Grade 5.8 10 mm	Grade 5.8 12 mm	Grade 8.8 10 mm	Grade 8.8 12 mm
*f_yb_* [MPa]	399.0	485.5	842.0	850
*f_ub_* [MPa]	483.0	564.0	935.0	908

**Table 3:** The results of the push-out tests of the shear connections with 10-mm grade 8.8 bolts and without toothed-plate connectors (per one connector).

Parameter	Specimen				Mean (R8.8.10.1–R8.8.10.4)
	R8.8.10.1	R8.8.10.2	R8.8.10.3	R8.8.10.4	
*P_ult_* [kN]	37.4	35.5	35.3	33.7	35.5 ± 2.4 (6.8%)
*s_ult_* [mm]	47.6	48.6	49.0	46.9	48.0 ± 1.5 (3.2%)
*k*_0.4_ [kN/mm]	5.6	5.3	5.6	5.3	5.5 ± 0.3 (5.1%)
*k*_0.6_ [kN/mm]	3.8	4.0	3.9	4.6	4.1 ± 0.6 (14.0%)

**Table 4:** The results of the push-out tests of the shear connections with 10-mm grade 8.8 bolts and with toothed-plate connectors (type C2-50/M10G, Bulldog) (per one connector).

Parameter	Specimen				Mean (8.8.10.1–8.8.10.4)
	8.8.10.1	8.8.10.2	8.8.10.3	8.8.10.4	
*P_ult_* [kN]	37.8	38.1	36.2	37.4	37.4 ± 1.3 (3.6%)
*s_ult_* [mm]	47.4	42.7	47.4	36.7	43.6 ± 8.1 (18.5%)
*k*_0.4_ [kN/mm]	6.0	4.8	7.5	4.7	5.8 ± 2.1 (36.2%)
*k*_0.6_ [kN/mm]	6.7	4.9	7.3	5.1	6.0 ± 1.9 (31.4%)

**Table 5:** The results of the push-out tests of the shear connections with 12-mm grade 8.8 bolts and without toothed-plate connectors (per one connector).

Parameter	Specimen				Mean (R8.8.12.1–R8.8.12.4)
	R8.8.12.1	R8.8.12.2	R8.8.12.3	R8.8.12.4	
*P_ult_* [kN]	38.2	37.3	36.8	38.3	37.7 ± 1.2 (3.1%)
*s_ult_* [mm]	45.5	46.9	46.8	44.4	45.9 ± 1.9 (4.1%)
*k*_0.4_ [kN/mm]	9.2	5.8	10.0	7.0	8.0 ± 3.1 (38.6%)
*k*_0.6_ [kN/mm]	7.8	5.5	7.9	5.9	6.8 ± 2.0 (29.4%)

**Table 6:** The results of the push-out tests of the shear connections with 12-mm grade 8.8 bolts and with toothed-plate connectors (type C2-50/M12G, Bulldog) (per one connector).

Parameter	Specimen				Mean (8.8.12.1–8.8.12.4)
	8.8.12.1	8.8.12.2	8.8.12.3	8.8.12.4	
*P_ult_* [kN]	39.1	38.2	38.0	40.4	38.9 ± 1.7 (4.5%)
*s_ult_* [mm]	40.9	44.1	44.4	38.8	42.1 ± 4.3 (10.2%)
*k*_0.4_ [kN/mm]	8.3	6.0	9.2	10.6	8.5 ± 3.1 (36.0%)
*k*_0.6_ [kN/mm]	8.0	6.3	8.7	9.8	8.2 ± 2.3 (28.5%)

**Table 7:** The results of the push-out tests of the shear connections with 10-mm grade 5.8 bolts and without toothed-plate connectors (per one connector).

Parameter	Specimen				Mean (R5.8.10.1–R5.8.10.4)
	R5.8.10.1	R5.8.10.2	R5.8.10.3	R5.8.10.4	
*P_ult_* [kN]	30.7	30.6	29.8	29.8	30.2 ± 0.8 (2.6%)
*s_ult_* [mm]	33.1	29.4	32.6	32.3	31.9 ± 8.3 (2.7%)
*k*_0.4_ [kN/mm]	4.8	5.7	5.2	3.9	4.9 ± 1.2 (24.7%)
*k*_0.6_ [kN/mm]	2.1	3.1	2.7	1.7	2.4 ± 1.0 (41.2%)

**Table 8:** The results of the push-out tests of the shear connections with 10-mm grade 5.8 bolts and with toothed-plate connectors (type C2-50/M10G, Bulldog) (per one connector).

Parameter	Specimen				Mean (5.8.10.1–5.8.10.4)
	5.8.10.1	5.8.10.2	5.8.10.3	5.8.10.4	
*P_ult_* [kN]	24.7	25.3	23.8	25.9	24.9 ± 1.4 (5.7%)
*s_ult_* [mm]	19.7	20.9	20.1	20.3	20.3 ± 0.8 (3.9%)
*k*_0.4_ [kN/mm]	6.0	6.3	5.4	6.3	6.0 ± 0.7 (11.3%)
*k*_0.6_ [kN/mm]	6.9	7.0	6.5	7.0	6.9 ± 0.4 (5.5%)

**Table 9:** The results of the push-out tests of the shear connections with 12-mm grade 5.8 bolts and without toothed-plate connectors (per one connector).

Parameter	Specimen				Mean (R5.8.12.1–R5.8.12.4)
	R5.8.12.1	R5.8.12.2	R5.8.12.3	R5.8.12.4	
*P_ult_* [kN]	43.6	39.1	41.3	40.5	41.1 ± 3.0 (7.3%)
*s_ult_* [mm]	47.4	48.4	47.6	41.1	46.1 ± 5.4 (11.7%)
*k*_0.4_ [kN/mm]	5.5	5.1	10.0	4.5	6.3 ± 4.0 (63.8%)
*k*_0.6_ [kN/mm]	4.5	4.8	6.7	4.6	5.2 ± 1.7 (32.2%)

**Table 10:** The results of the push-out tests of the shear connections with 12-mm grade 5.8 bolts and with toothed-plate connectors (type C2-50/M12G, Bulldog) (per one connector).

Parameter	Specimen				Mean (5.8.12.1–5.8.12.4)
	5.8.12.1	5.8.12.2	5.8.12.3	5.8.12.4	
*P_ult_* [kN]	42.5	39.6	40.2	38.0	40.1 ± 3.0 (7.4%)
*s_ult_* [mm]	51.4	38.5	43.1	35.0	42.0 ± 11.3 (26.9%)
*k*_0.4_ [kN/mm]	5.6	10.0	8.8	4.7	7.3 ± 4.0 (55.3%)
*k*_0.6_ [kN/mm]	5.2	8.3	7.8	5.4	6.7 ± 2.6 (38.2%)

**Table 11:** A comparison of the obtained results with the literature [49].

Connection	Connector Dimensions [mm]	Connector Grade	Reinforcing Toothed Plate	*P_ult_* [kN]	*s_ult_* [mm]	*k*_0.4_ [kN/mm]	*k*_0.6_ [kN/mm]
*Screwed*	10 × 80	5.8	–	16.7	16.7	4.3	4.7
*Screwed*	10 × 80	5.8	C2-50/M10G	21.5	12.8	6.4	5.9
*Screwed*	12 × 80	5.8	–	22.3	24.0	8.5	7.1
*Screwed*	12 × 80	5.8	C2-50/M12G	27.6	12.8	7.5	7.3
*Bolted*	10 × 125	5.8	–	30.2	31.9	4.9	2.4
*Bolted*	10 × 125	5.8	C2-50/M10G	24.9	20.3	6.0	6.9
*Bolted*	12 × 135	5.8	–	41.1	46.1	6.3	5.2
*Bolted*	12 × 135	5.8	C2-50/M12G	40.1	42.0	7.3	6.7

## Data Availability

All data are contained within the article.
